# Percutaneous transhepatic cholangioscopy with cholangiography-guided choledochojejunostomy: a bridge drainage technique for distal common bile duct stenosis after Begerʼs operation

**DOI:** 10.1055/a-2566-9510

**Published:** 2025-04-10

**Authors:** Rui Chen, Jingyi Zhang, Tianhao Chen, Jie Zhang, Rongxing Zhou

**Affiliations:** 1 34753 Division of Biliary Surgery, Department of General Surgery, West China Hospital of Sichuan University, Chengdu, China; 2 34753 Research Center for Biliary Diseases, West China Hospital of Sichuan University, Chengdu, China; 3 34753 Department of Ultrasound, West China Hospital of Sichuan University, Chengdu, China

A 48-year-old woman who had undergone Beger's operation for a serous cystic adenoma of the pancreatic head 5 months previously, suffered recurrent fever and jaundice for 3 months. Abdominal computed tomography and two failed attempts at endoscopic retrograde cholangiopancreatography (ERCP) indicated a distal common bile duct (CBD) stenosis ( Fig. 1 ). The bridge drainage technique applied between two non-communicating anatomic structures is potentially a promising biliary drainage strategy for malignant or benign biliary obstruction 1 2 3 . Given the Roux-en-Y limb of pancreaticojejunostomy was adjacent to the CBD after the Begerʼs operation and to minimize duodenal biliary reflux, we report for the first time a novel bridge drainage approach between the CBD and the adjoining Roux-en-Y limb using percutaneous transhepatic cholangioscopy, instead of recanalization of the CBD stenosis ( Video 1 ).

**Fig. 1:**
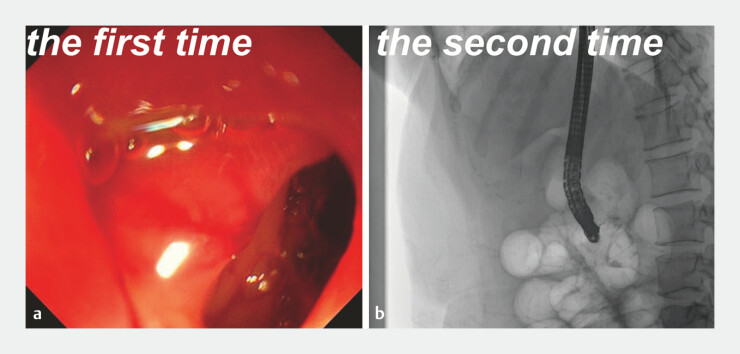
Images from two endoscopic retrograde cholangiopancreatographies that failed: **a** owing to the duodenal stenosis; **b** with biliary cannulation proving impossible.

Bridging of the common bile duct (CBD) and the adjoining Roux-en-Y limb using ultrasound-guided percutaneous transhepatic cholangioscopy is successfully performed to treat a distal CBD stenosis after Begerʼs operation.Video 1

The operation started with ultrasound-guided one-step puncture of the CBD. A guidewire was inserted, facilitating the use of dilators until an 18-Fr sheath could be placed. Multiple attempts to pass the guidewire through the CBD stenosis were unsuccessful ( Fig. 2 ). In situ puncture towards the left and ventral side in the established sheath was then performed under ultrasound guidance to link the CBD with the Roux-en-Y limb. The puncture channel was further expanded by a ballon, with good flow of contrast medium from the CBD into the bowel ( Fig. 3 ). Finally, an 18-Fr biliary drainage catheter was placed for support and drainage, with a 10-Fr pigtail catheter placed inside to prevent distal displacement ( Fig. 4 **a** ).

**Fig. 2:**
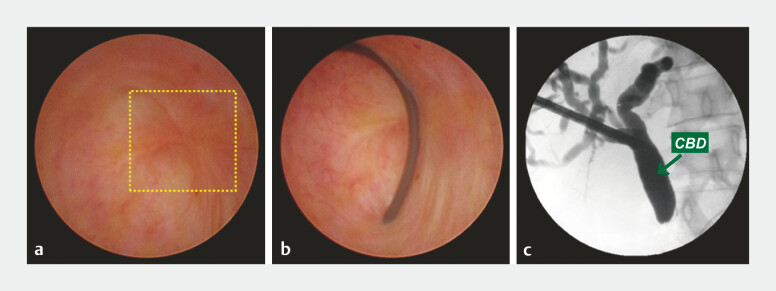
Direct cholangioscopic visualization and intraoperative cholangiography showing: **a** severe distal common bile duct (CBD) stenosis, with a completely obstructed CBD (yellow dashed square); **b** a failed attempt to pass the guidewire through the CBD stenosis; **c** no passage of contrast medium into the bowel system on cholangiography.

**Fig. 3:**
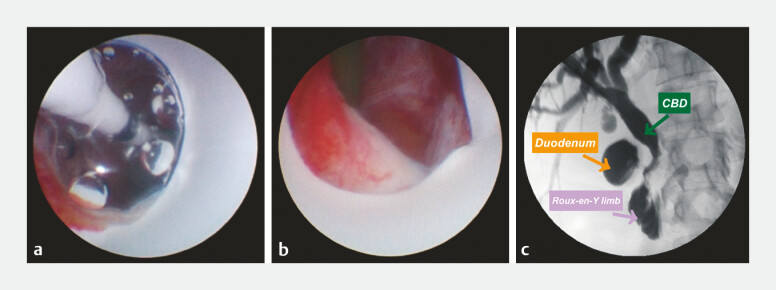
Successful creation of the connection between the common bile duct (CBD) and the adjoining Roux-en-Y limb showing: **a** the biliary ballon placed through the puncture approach and repeatedly inflated to a pressure of 10 bar for 2 minutes to dilate the channel; **b** the successfully dilated puncture channel; **c** the flow of contrast medium from the CBD into the bowel system on cholangiography.

**Fig. 4:**
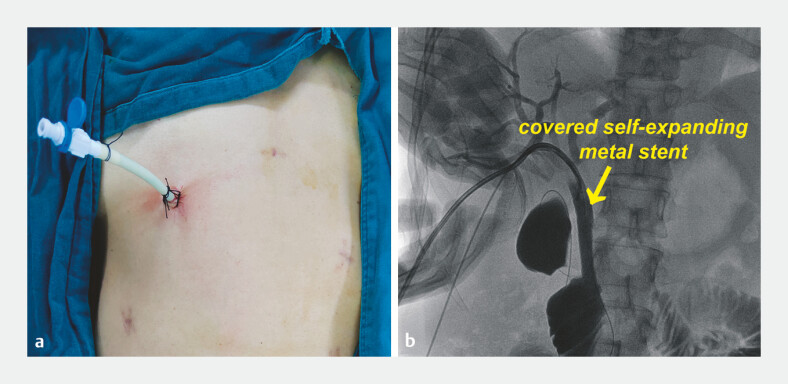
Postoperative appearance: **a** initially, with an 18-Fr biliary drainage catheter positioned in the common bile duct (CBD) for support and drainage, and a 10-Fr pigtail catheter inside the catheter to prevent distal displacement; **b** 4 weeks later, a covered self-expanding metal stent placed through the drainage catheter to achieve internal drainage under percutaneous fluoroscopic guidance.

After 4 weeks, during which there were no postoperative complications, a covered self-expanding metal stent was placed under percutaneous cholangiography guidance as a replacement for the drainage catheter to bridge the CBD and the adjoining Roux-en-Y limb, thereby achieving completely internal drainage ( Fig. 4 **b** ). Ultrasound-guided percutaneous transhepatic cholangioscopy combined with cholangiography for bridging of the CBD and the adjoining Roux-en-Y limb, while minimizing duodenal biliary reflux, may provide a safe and feasible treatment option for such CBD stenosis after failed ERCP.

Endoscopy_UCTN_Code_TTT_1AR_2AI

## Notes

Endoscopy E-Videos https://eref.thieme.de/e-videos
*E-Videos* is an open access online section of the journal *Endoscopy* , reporting on interesting cases and new techniques in gastroenterological endoscopy. All papers include a high-quality video and are published with a Creative Commons CC-BY license. Endoscopy E-Videos qualify for HINARI discounts and waivers and eligibility is automatically checked during the submission process. We grant 100% waivers to articles whose corresponding authors are based in Group A countries and 50% waivers to those who are based in Group B countries as classified by Research4Life (see: https://www.research4life.org/access/eligibility/ ). This section has its own submission website at https://mc.manuscriptcentral.com/e-videos .
